# Impact of internal mammary artery perforator propeller flaps combined with radiotherapy in the treatment of large chest keloids: Our experience

**DOI:** 10.3389/fsurg.2023.1136496

**Published:** 2023-04-04

**Authors:** Jianfang Zhao, Kun Xie, Shangbin Qin, Rui He, Shan Jiang, Xin Qi, Bing Wen

**Affiliations:** ^1^ Department of Plastic and Burn Surgery, Peking University First Hospital, Beijing, China; ^2^ Department of Radiotherapy, Peking University First Hospital, Beijing, China

**Keywords:** keloids, internal mammary artery perforator flaps, radiotherapy, scar management, reconstruction

## Abstract

**Background:**

Keloids are benign skin hyperplasias but have a tumor-like appearance. Clinical management of keloids remains challenging.

**Aims:**

We retrospectively evaluated the safety and efficacy of internal mammary artery perforator propeller flaps combined with timely radiotherapy in the treatment of large chest keloids.

**Methods:**

From June 2017 to May 2020, 25 patients with large chest keloids (average size 4.82 cm ± 2.53 cm × 9.04 cm ± 4.86 cm) who received both radiotherapy and internal mammary artery perforator flaps transplantation in our department were included. After surgical removal of the keloids, various propeller flaps based on the unilateral internal mammary artery were designed and applied to repair the defects. Timely and full-dose radiotherapy was performed for these patients after the operation.

**Results:**

After keloid resection, the dimensions of the defect area were 3 cm–15 cm × 4 cm–25 cm, and the sizes of the flaps were 3 cm–16 cm × 4 cm–27 cm. For all 25 patients, the flaps survived, and the incisions healed in one stage. During the follow-up (median 18 months), no local recurrence was observed, and the itching and pain symptoms in the scar area were significantly relieved. Both physicians and patients were satisfied with the results.

**Conclusions:**

The application of internal mammary artery perforator propeller flaps combined with radiotherapy in the treatment of chest keloids can effectively reduce the recurrence of keloids and relieve the related symptoms. It also has advantages including minimized donor site damage, short operation time and speedy postoperative recovery, suggesting its great clinical value.

## Introduction

Keloids are the result of excessive proliferation of collagen fibers due to the loss of normal control of collagen synthesis and metabolism during skin injury healing (1). They usually occur in regions with relatively high tension, such as the parasternal area, shoulders and upper back. The common causes of keloids are trauma, surgery and infection (2). Although keloids are benign hyperplasias, their clinical manifestations, such as pain, pruritus and ulceration, often cause functional impairment, and their tumor-like appearance may lead to psychosocial morbidity and seriously affect the quality of life (3). Therapeutic options for keloids include surgery, radiation and other nonsurgical treatments. For large keloids, the effects of nonsurgical therapies such as corticosteroid injection, pressure therapy, laser treatment and silicone gel dressing are limited (4). The literature has shown that surgical excision combined with postoperative radiation could result in an ideal control rate for large keloids (5). For chest wall keloid management, Long et al. mentioned the application of several flaps for reconstruction after resection, including local flaps, internal mammary artery perforator (IMAP) flaps and keystone-designed island flaps (6, 7). In the current study, we introduced our experience in using IMAP propeller flaps for reconstructing the large defect left by chest keloid excision, followed by radiotherapy using an electron beam. The outcome was satisfactory.

## Materials and methods

### Patient information

This retrospective study was approved by the local ethics committee and conformed to the World Medical Association Declaration of Helsinki. All patients signed informed consent forms and agreed to the use of their imaging for medical research.

From June 2017 to May 2020, 27 patients with chest wall keloids were treated in our department. Twenty-five patients who received both radiotherapy and IMAP flaps transplantation were included in this retrospective study. Among these 25 patients, 8 were males, and 17 were females, aged from 24 to 79 years, with a median age of 63 years. The causes of the keloids were surgery for 12 patients, trauma for 7 patients and folliculitis infection for 6 patients. The sizes of the keloids were 3 cm–15 cm × 4 cm–25 cm, with an average size of 4.82 cm ± 2.53 cm × 9.04 cm ± 4.86 cm. The average course of the keloids was 21.6 months. All patients complained of different degrees of pain and itching, and 16 patients had recurrent ulceration and localized infection before the operation. Detailed information on the 25 patients is listed in Table 1.

**Table 1:** Detail information of 25 patients.

Gender	Age (year)	Cause of keloids	Course of keloids (month)	Size of keloids (cm × cm)	Ulceration and infection	Previous treatment	Size of defect (cm × cm)	Perforator	Size of flap (cm × cm)	Follow-up duration (month)
Male	66	Trauma	12	4 × 3	No	Intralesional steroids	3 × 3	Left second intercostal	4 × 3	14
Female	28	Surgery	6	4 × 3	Yes	None	4 × 4	Right fourth intercostal	4 × 4	15
Female	42	Infection	21	4 × 3	Yes	Intralesional steroids	4 × 3	Left fifth intercostal	4 × 4	18
Male	35	Surgery	15	5 × 4	Yes	None	5 × 4	Left second intercostal	5 × 5	24
Female	67	Surgery	23	5 × 3.5	No	None	6 × 5	Left fifth intercostal	6 × 6	12
Female	46	Infection	36	4 × 4	No	None	7 × 5	Right third intercostal	8 × 6	18
Female	38	Surgery	11	6 × 3	Yes	Intralesional steroids	7 × 3	Right fifth intercostal	7 × 5	21
Female	65	Surgery	16	6 × 4.5	No	None	7 × 5	Left second intercostal	7 × 6	30
Female	79	Surgery	9	7 × 6	Yes	None	8 × 6	Left second intercostal	10 × 8	15
Female	66	Trauma	42	5 × 4.5	Yes	Excision and compression	8 × 6	Left second and third intercostal	13 × 8	32
Female	65	Surgery	24	9 × 4	No	None	9 × 6	Right fifth intercostal	12 × 7	12
Female	63	Infection	30	8 × 3	Yes	Intralesional steroids	9 × 5	Right fourth intercostal	10 × 8	15
Female	51	Surgery	18	12 × 3	Yes	Intralesional steroids	9 × 5	Left fifth intercostal	9 × 6	20
Male	56	Trauma	24	9 × 7	No	Intralesional steroids	9 × 4.5	Left second intercostal	10 × 5	21
Male	24	Infection	36	10 × 8	Yes	Intralesional steroids	10 × 8	Right second intercostal	12 × 10	18
Male	64	Trauma	15	15 × 3	Yes	None	11 × 6	Left third intercostal	12 × 7	12
Female	53	Trauma	36	7 × 6	Yes	Intralesional steroids	12 × 8	Left second intercostal	13 × 9	18
Male	57	Trauma	12	12 × 6	Yes	None	12 × 6	Left second intercostal	13 × 8	25
Female	35	Trauma	10	10 × 4	Yes	None	12 × 4	Right fifth intercostal	14 × 4	27
Male	70	Surgery	20	12 × 6	No	Intralesional steroids	12 × 6	Left third intercostal	14 × 6	28
Female	66	Infection	25	9 × 4	Yes	Intralesional steroids	15 × 9	Left fourth intercostal	16 × 12	24
Female	40	Surgery	34	15 × 4	Yes	Excision and compression	16 × 5	Left second intercostal	16 × 6	12
Female	63	Surgery	21	8 × 5	No	Intralesional steroids	16 × 7	Left second intercostal	18 × 8	32
Male	79	Surgery	20	15 × 4	Yes	Intralesional steroids	20 × 5	Right second intercostal	22 × 5	21
Female	78	Infection	24	25 × 15	No	Intralesional steroids	25 × 5	Right fifth and sixth intercostal	27 × 16	16

### Surgical technique

**Figure 1:**
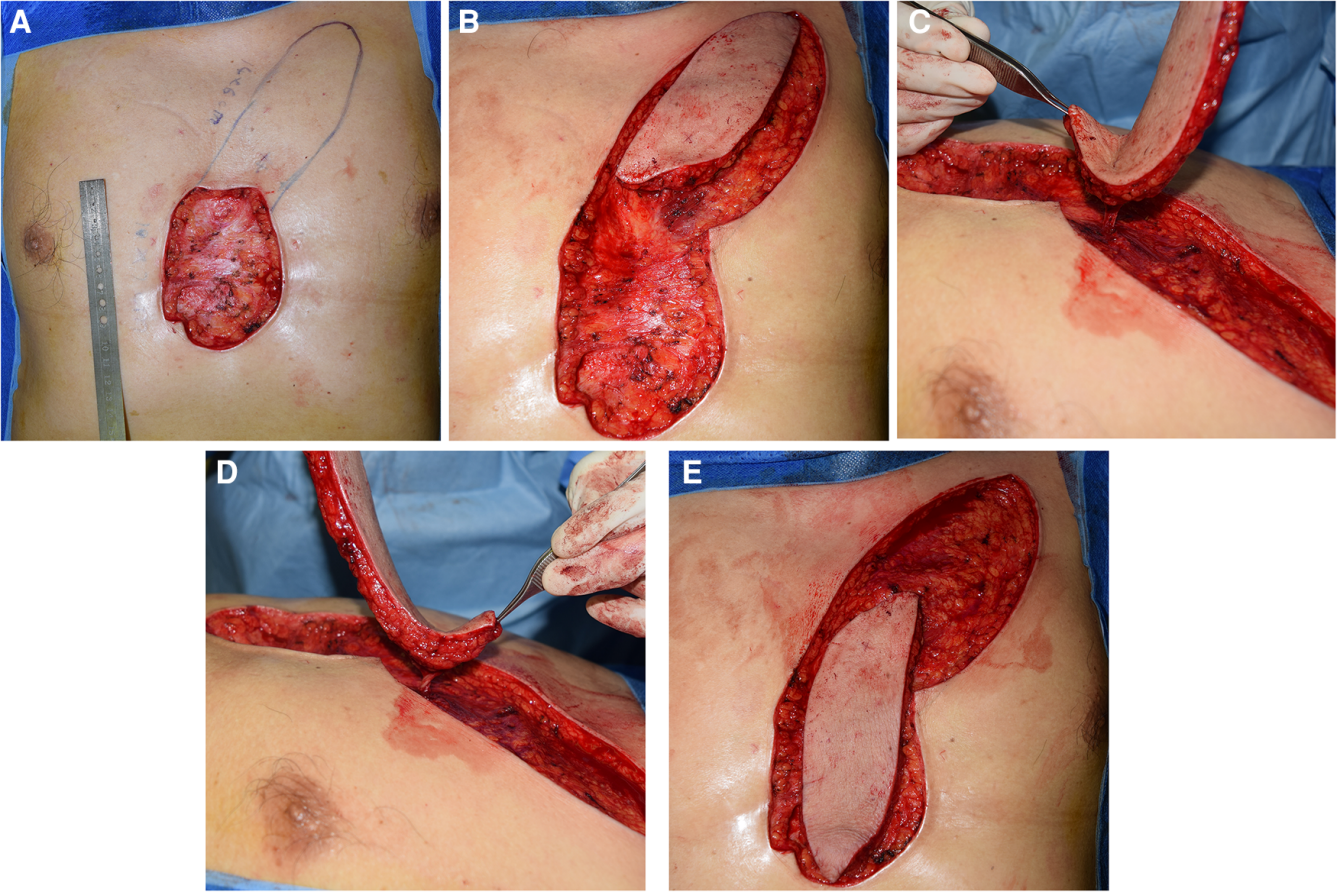
Surgical procedures. (**A**) Measured and designed the flap according to the shape and size of the defect. (**B,C**) The flap was dissected and the vascular pedicle was carefully freed to ensure rotation. (**D,E**) The flap was rotated and covered the defect without much tension.

The positions of perforating vessels of the internal mammary artery were detected and marked using color Doppler ultrasound before the operation. The operation was performed under general anesthesia. A circular incision was made 1 mm away from the edge of keloids, the skin and subcutaneous tissue were cut, and the keloids in the superficial layer of deep fascia were completely removed. After complete hemostasis, the wound was rinsed with normal saline. The shape and size of the defect area that could not be sutured by primary closure were carefully measured. The perforator was detected again using a hand-held Doppler device, and the rotation point was determined according to the position of the vessels. As shown in Figure 1, the IMAP flap was designed according to the shape of the defect so that the distance between the distal end of the defect and the rotation point was equal to the distance between the distal end of the flap and the rotation point. The distal part of the flap was dissected in the superficial fascia layer and gradually transferred to the deep fascia layer after it was placed close to the vascular pedicle. Hand-held Doppler ultrasound was applied repeatedly to confirm the position of the perforator during this process. The vascular pedicle was carefully freed to an adequate length to ensure that the flap had enough mobility to completely cover the defect without much tension. The skin incision was closed using tension reduction sutures, and a drainage tube was placed under the flap.

### Radiotherapy

Within 24 h after keloid resection, both the donor site and recipient site were treated with electron beam irradiation using a VARIAN2300CD electron linear accelerator (VARIAN company, USA). After protecting the normal skin around the incision with a lead plate, 6 MeV electron beam irradiation was delivered 0.5 cm–1.0 cm away from the surgical incision (including the needle eye of the suture) once a day for 5 consecutive days as a course of treatment, with a dose of 5 Gy each time and a total dose of 25 Gy.

### Postoperative care and effect assessment

The blood supply of the flap was closely observed after the operation. The drainage tube was removed when the drainage volume was less than 10 ml per day, generally 2–3 daysafter surgery. Sutures were removed 14 days after the operation, and silicone gel sheets were applied for 6–12 months.

The median follow-up duration was 18 months (range from 12 to 32 months). The Patient and Observer Scar Assessment Scale (POSAS) was used before and after surgery to assess the treatment effect (8). Local recurrence of keloids was also determined using the following criteria: scar hyperplasia exceeded the original surgical incision, with or without symptoms such as scar itching and pain.

### Statistical analysis

SPSS (version 22.0, SPSS Inc., Chicago, USA) was used for data processing, analysis and calculation. The Kolmogorov‒Smirnov method was used to test whether each group of data followed a normal distribution. Data with a normal distribution are expressed as the mean ± standard deviation $\left(\bar{x}\pm \mathrm{SD}\right)$ and were compared with the independent-samples *t* test. Data that did not follow a normal distribution are expressed as the median. Counting data are expressed as percentages (%). A *P* value < 0.05 was considered statistically significant and is marked with * in the table.

## Results

After keloid resection, the size of the defect area was 3 cm–15 cm × 4 cm–25 cm, with an average size of 5.74 cm ± 2.46 cm × 10.24 cm ± 5.16 cm. The size of the flaps was 3 cm–16 cm × 4 cm–27 cm, with an average dimension of 6.88 cm ± 2.80 cm × 11.44 cm ± 5.63 cm. All donor sites and recipient sites were closed without tension. All flaps survived and healed without complications.

**Figure 2:**
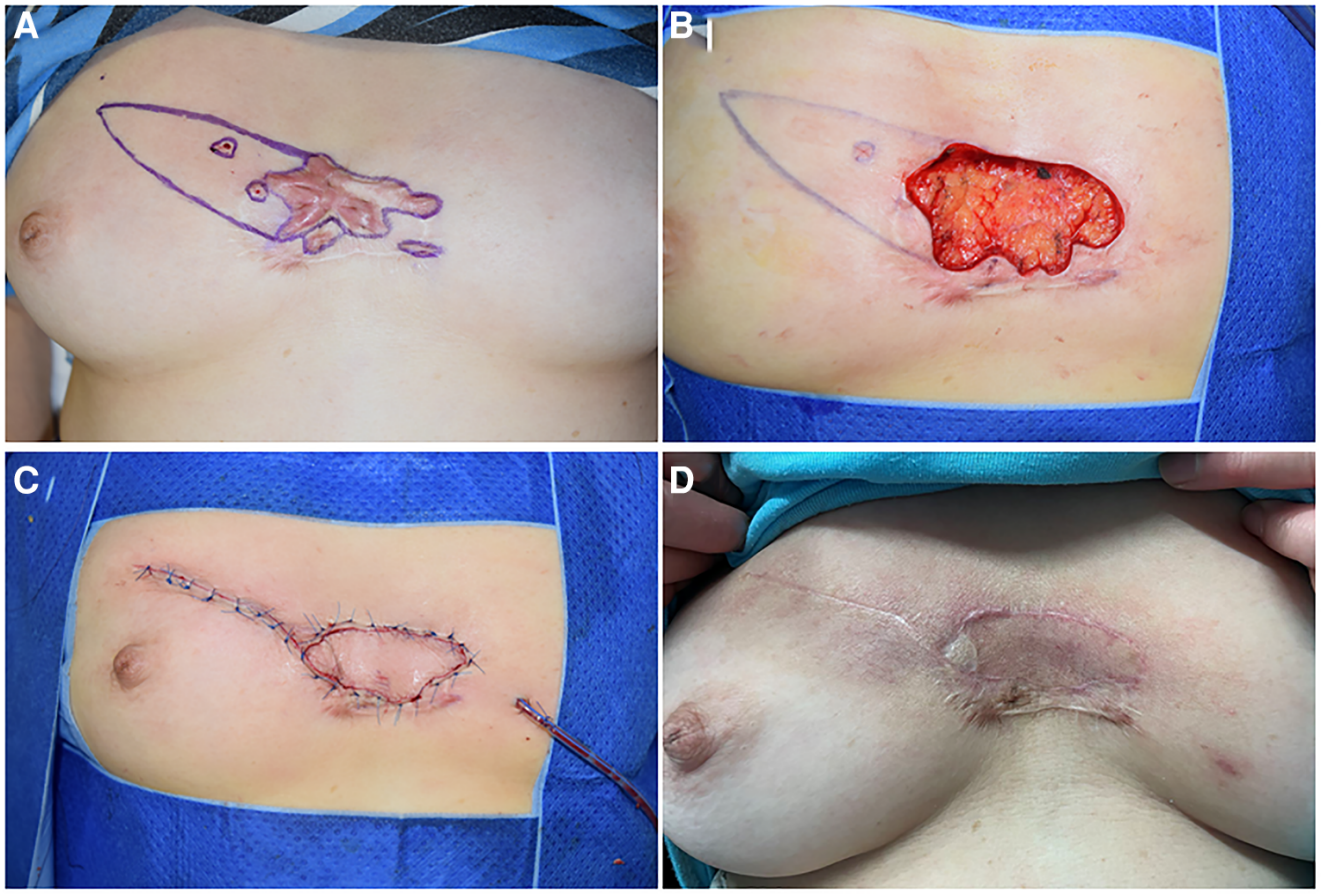
A 46-year-old female suffering from anterior chest wall keloids. (**A**) The position of the perforators was detected, and the resection areawas marked. (**B**) A defect of 5 cm × 7 cm is left after the resection. The flap was designed, and the vessel was detected repeatedly during detachment of the flap. (**C**) The defect was covered without tension. (**D**) 18 months after the operation.

**Figure 3:**
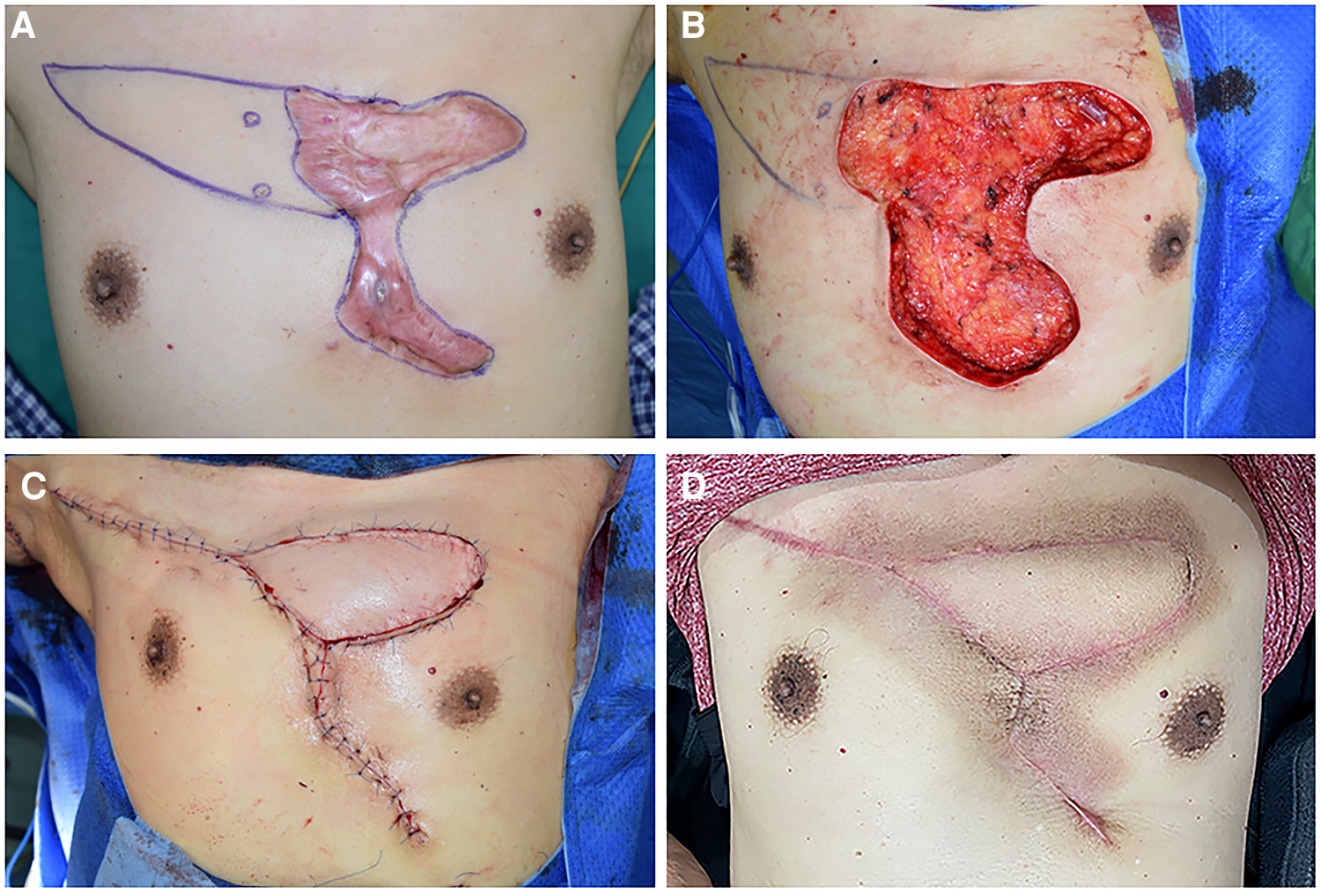
A 57-year-old male suffering from a large anterior chest wall keloid that had recurrent ulceration. (**A**) The position of the perforators was detected, and the resection area was marked. (**B**) The size of the upper portion of the defect area that could not be sutured by primary closure was 6 cm × 12 cm and was left after resection. (**C**) An IMAP flap was applied to cover the defect, and the skin incision was closed without much tension. (**D**) 15 months after the operation.

**Figure 4:**
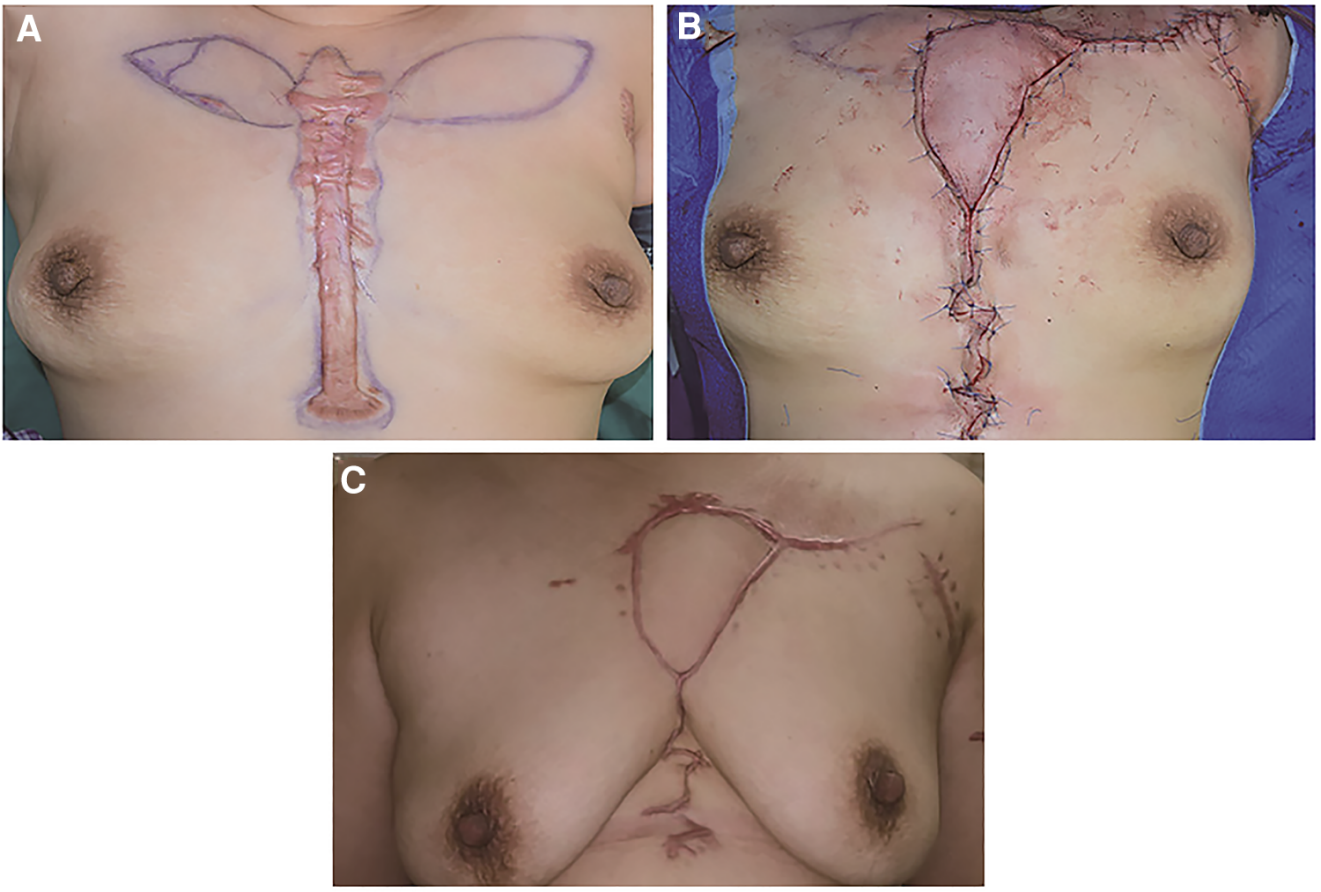
A 60-year-old female suffering from an anterior chest wall keloid. (**A**) The bilateral IMAP was detected, and its position was marked. (**B**) A left side IMAP flap was chosen to cover the defect. (**C**) 12 months after the operation.

No keloid recurrence was observed during the follow-up. Among the 25 patients, 2 experienced local epidermal exfoliation, which recovered after 4–6 months. The POSAS evaluation results are shown in Table 2. Both the doctor score and patient score after surgery were significantly lower than those before surgery, indicating satisfactory treatment results. Typical cases are shown in Figures 2–4.

**Table 2:** The patient and observer scar assessment scale results.

	Before surgery	During follow-up	*P* value
Physician sore (total 50)	28.75 ± 2.38	9.56 ± 2.11	<0.001^*^
Patient score (total 60)	36.27 ± 5.15	13.23 ± 4.02	<0.001^*^

## Discussion

Keloids are one of the most challenging problems in clinical treatment. The mechanism of keloid occurrence has not been fully illustrated but is considered to be related to internal factors such as heredity, race, age and skin tension and external factors such as injury, infection and suture technique (1). Keloids likely occur in high tension areas such as the chest wall, suggesting that their occurrence is highly associated with the tension of the wound. Although many treatment methods have been developed, the recurrence rate of keloids is still high. Surgical resection combined with radiotherapy has been shown to have the lowest recurrence rate (4).

For large keloids located in high tension areas, plastic and reconstructive techniques are often needed to repair the defect left by surgical resection. The tissue expansion technique has been widely used since the 1970s. It can avoid donor site injury and ensure similar color and texture between flaps and recipient area (9). The application of tissue expander in treating giant keloids has also been described (10). However, tissue expansion has a long therapeutic process with several complications. One study used scar-centered dilation to treat body keloids and two patients who had anterior chest keloids occurred chest incision broken after the surgery (11). A few studies suggested free skin grafts in large keloids treatment (12, 13). However, skin grafts can lead to new donor site scars and long-term skin contraction and pigmentation. Therefore, a skin flap is undoubtedly the best choice for one-stage tension-free reconstruction following keloid resection.

In recent years, the application of perforator flaps has gradually increased. This kind of flap belongs to the category of axial vascular flaps and relies on the blood supply of a large variety of perforating vessels, even those with small diameters (14). Perforator flaps can be harvested from multiple areas outside the conventional donor sites of myocutaneous flaps. Moreover, tissues can be customized and trimmed efficiently according to the demand. The application of perforator flaps allows a flexible design and precise dissection, minimizes donor sitedamage and eventually achievesexcellent aesthetic appearance and function (15). The IMAP flap, which refers to an axial flap pedicled with the perforators of the internal mammary artery, is an ideal choice for the reconstruction of chest wall defects. The bilateral internal mammary arteries originate from the subclavian artery, enter the chest cavity downward, and descend vertically to the inner surface of the chest wall approximately 1 cm away from the outer edge of the sternum (16). There may be perforating branches between the first and sixth intercostal arteries. Commonly, the second intercostal perforator is relatively thick and constant, and the corresponding flap is widely used in head and neck reconstruction (17). During our clinical practice, we noticed that there was a large amount of tissue near the inframammary fold in female patients, so the flaps pedicled with the fifth and sixth intercostal perforators were often designed to hide the donor site scar and reduce the tension of the donor area. Since most of the keloids on the chest wall were located on the surface of the sternum, the IMAP propeller flaps could easily repair the defect, and both the donor and recipient sites could simultaneously undergo radiotherapy, minimizing pain and shortening the recovery time. Due to the difference in the diameter of IMAPs, color Doppler ultrasound was used to determine the vessels before the operation, and hand-held Doppler ultrasound was applied repeatedly during the operation to select the best perforator and ensure successful treatment.

Surgical resection combined with postoperative radiotherapy can effectively treat keloids. Within 24 h after the operation, the vast majority of cells in the wound granulation tissue are fibroblasts, which are highly sensitive to radiation but gradually become fibrocytes. Therefore, the earlier the application of radiotherapy to inhibit the formation of fibroblasts, the less the growth and recurrence of keloids. In addition, postoperative radiotherapy can inhibit the stimulation of immune cells to fibroblasts, reduce the synthesis of collagen fibers and control the formation of scars (18). At present, there is no standard recommendation for the dose of postoperative radiotherapy. However, evidence has indicated that the bioequivalent dose (BED) of postoperative radiotherapy for keloids should reach 30 Gy (19). One study showed that the local recurrence rate of keloids after 18 Gy/3 times (BED: 28.8 Gy) postoperative radiotherapy was 5.3% (20). Another group used 15 Gy/3 times (BED: 22.5 Gy) radiotherapy and obtained a recurrence rate of 26.7% (21). In the current study, we used 20 Gy/5 times (BED: 28 Gy) *via* electron beam irradiation. The median follow-up was 18 months, and there was no local recurrence. The late sideeffects of postoperative radiotherapy include pigmentation, depigmentation, skin atrophy and skin ulcers. As of the follow-up date, no significant late side effects have occurred in any of the patients.

The limitation of using IMAP propeller flaps to treat chest keloids is that for patients underwent thoracotomy, or patients with chest tumor who received radiotherapy after primary surgery, their internal mammary arteries or perforating vessels may have been damaged and could not supply the propeller flaps. In this case, other method should be considered. This approach is especially suitable for older women who have relatively loose and sagging breasts.

In conclusion, the application of the IMAP propeller flap combined with radiotherapy in the treatment of large chest keloids has advantages including minimized donor site damage, short operation time, speedy postoperative recovery, low recurrence rate and good functional outcome and has good clinical application prospects.

## Data Availability

The original contributions presented in the study are included in the article/Supplementary Material, further inquiries can be directed to the corresponding author.
